# Exploring the relationship between exercise and diabetes risk in a prediabetes population

**DOI:** 10.7150/ijms.116287

**Published:** 2025-09-22

**Authors:** Chih-Wei Chiang, Oswald Ndi Nfor, Wen-Yu Lu, Chien-Ning Huang, Yung-Po Liaw

**Affiliations:** 1 Department of Public Health and Institute of Public Health, Chung Shan Medical University, Taichung City 40201, Taiwan.; 2 College of Health Care and Management, Chung Shan Medical University, Taichung City 40201, Taiwan.; 3 Department for Nursing, Jen-Teh Junior College of Medicine, Nursing and Management, Miaoli County 456, Taiwan.; 4 Institute of Medicine, Chung Shan Medical University, Taichung City 40201, Taiwan.; 5 Department of Internal Medicine, Chung Shan Medical University Hospital, Taichung City 40201, Taiwan.; 6 Department of Medical Imaging, Chung Shan Medical University Hospital, Taichung City 40201, Taiwan.

**Keywords:** disease prevention, epidemiology, physical activity, risk

## Abstract

The global incidence of prediabetes is on the rise, with an estimated 5 to 10% of individuals expected to transition to diabetes. We investigated factors associated with the progression of prediabetes to diabetes. Our primary data source was the Taiwan Biobank (TWB). The main outcome was the development of diabetes during the follow-up period among individuals who were initially diagnosed with prediabetes. We included 4,958 participants from the TWB, who were divided into four groups based on their levels of exercise. The exercise status of participants was assessed based on questionnaire responses collected during the enrollment and follow-up phases. Participants were categorized into one of the following groups: no exercise, transition from no exercise to exercise, transition from exercise to no exercise, and regular exercise. We used multiple logistic regression to establish the analysis model, which comprised 2,891 women and 2,067 men. The exercise group, comprising individuals who consistently engaged in exercise both at enrollment and during the follow-up period, exhibited a lower risk of developing diabetes (odds ratio [OR] = 0.755; 95% confidence interval [CI] = 0.640-0.892) compared to the no exercise group. When stratified by gender, the exercise group remained significantly associated with a reduced risk of diabetes in both women (OR = 0.752, 95% CI = 0.602-0.940) and men (OR = 0.762, 95% CI = 0.591-0.982)**.** This study provides evidence of a significant association between maintaining regular exercise habits and a lower risk of diabetes among Taiwanese adults with prediabetes.

## Introduction

Individuals with prediabetes eventually develop diabetes, with roughly 10% transitioning to diabetes annually in the United States, although the conversion rates differ based on disease definition and population characteristics 1, 2. Prediabetes, an intermediate stage of glucose dysregulation that often precedes type 2 diabetes (T2D), affected around 720 million individuals globally in 2021 1, 3. By 2045, it is estimated to affect 1 billion people worldwide. T2D significantly elevates the risk of heart disease, stroke, and other severe cardiovascular health issues, contributing to disease mortality and morbidity 4. The rising prevalence of prediabetes on a global scale represents a significant public health issue and is concerning in light of the expanding diabetes epidemic and its associated complications 5.

Prediabetes is a state with elevated blood sugar levels that are higher than normal but not yet at the diabetes threshold, indicating a high risk for developing diabetes 2. It is a multifaceted metabolic condition characterized by several risk factors that mirror those associated with type T2D. These include family history, the presence of concurrent chronic conditions (such as hypertension and dyslipidemia), and behavioral risk factors 6. Among these, overweight, obesity, and dietary habits are the most predominant factors 1. In Taiwan, diabetes is one of the top ten causes of death, and in recent years, there has been an increasing trend in diabetes-related mortality. In the year 2022, the number of deaths due to diabetes increased by 7.3% compared to the previous year 7.

A meta-analysis revealed that individuals with prediabetes at baseline exhibited elevated mortality rates and increased incidence of cardiovascular events 8. Research conducted on diabetes prevention in China, Finland, and the United States reported annual progression rates from prediabetes to diabetes in their control groups, varying between 5.8% and 18.3% 9-11. Intensive lifestyle modifications and community mobilization against prediabetes can effectively lower diabetes incidence 12, 13.

According to the National Nutrition and Health Survey in Taiwan conducted between 2017 and 2020, approximately 25.5% of adults aged 19 and older have fasting blood sugar (FBS) levels that meet the prediabetic threshold 14*.* This threshold is defined as a FBS level ranging from 100 to 125 mg/dL, or 5.6 to 6.9 mmol/L. Approximately 35.8% (about 6.5 million people) of Taiwanese adults have FBS levels reaching the prediabetic threshold 15. A study in Taiwan suggested that when FBS reaches prediabetic levels, there is a significant increase in cardiovascular disease and diabetes mortality rates 16. Therefore, it is essential to prevent or treat prediabetes aggressively 17. Moreover, a healthy aging longitudinal study in Taiwan (HALST) found that individuals with prediabetes who had higher health scores (including diet, exercise, and psychological state) and a normal waist circumference had a lower risk of diabetes 18. To prevent the onset of diabetes, it is essential to implement targeted health behavior interventions for high-risk populations, including those with prediabetes.

To date, there have been relatively few studies in Taiwan that simultaneously examined prediabetes and modifiable behaviors, often with small sample sizes. This study aimed to investigate the factors associated with the progression of prediabetes to diabetes.

## Material and Methods

### Study population

**Figure 1:**
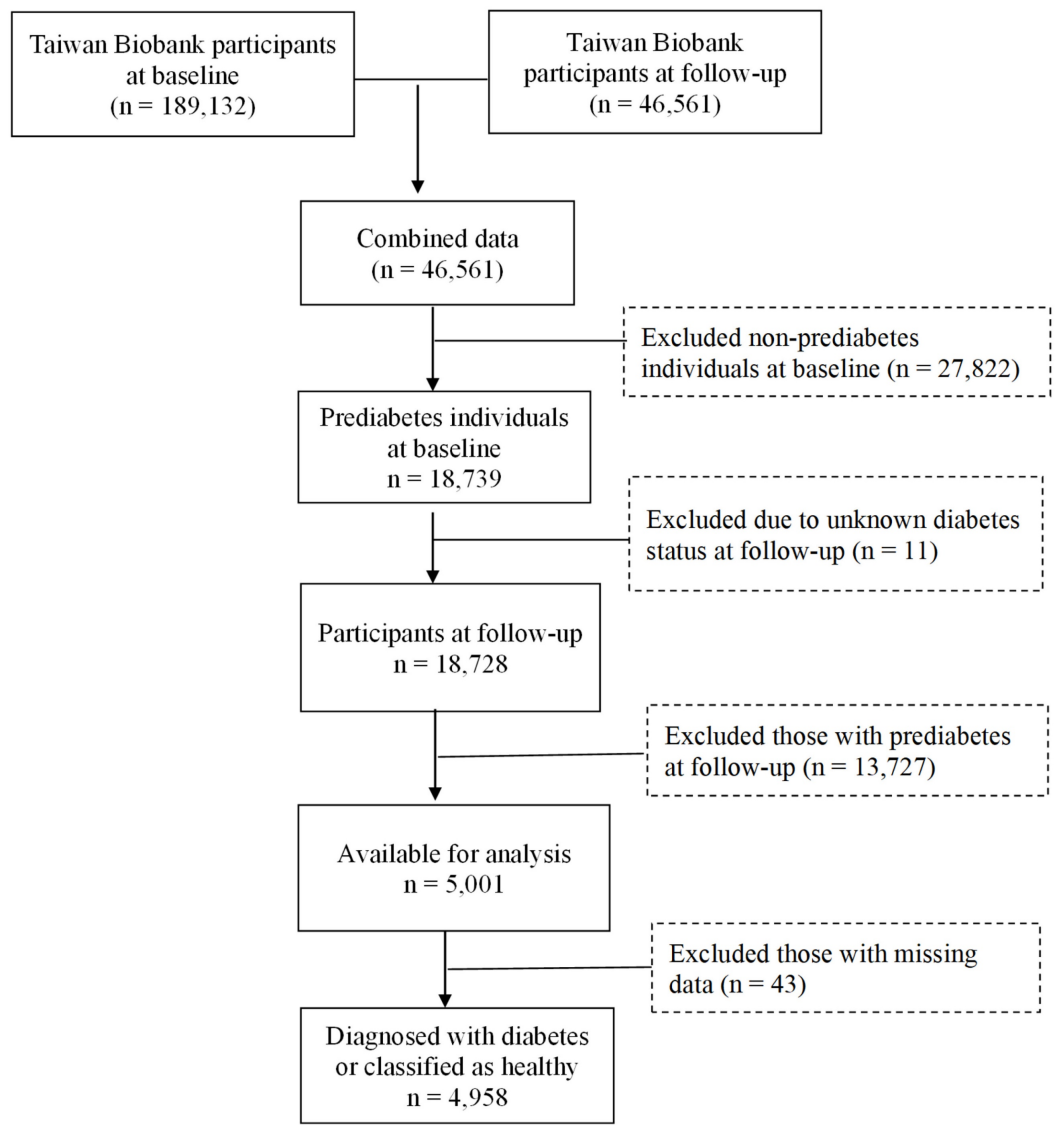
Flowchart of the study population.

Data for this study were obtained from the TWB, which is a voluntary repository for Taiwanese adults between the ages of 30 and 70, with no history of cancer. During enrollment, participants gave their written informed consent and underwent physical examinations at assessment centers. Blood and urine samples were collected for analysis. Lifestyle data were obtained through the biobank questionnaires. The current study focused on participants recruited from the Biobank between 2008 and 2019, with follow-up conducted from 2011 to 2022. The selection process is illustrated in Figure 1. Initially, 189,132 individuals were assessed at baseline, with 46,561 participants completing the follow-up. Among these 27,822 baseline non-prediabetes cases were excluded, leaving 18,739 individuals identified as having prediabetes. At follow-up, 13,727 individuals who still had prediabetes, as well as those with unknown diabetes status (n=11), were excluded, resulting in 5,001 individuals available for analysis. After removing 43 individuals with missing data, 4,958 remained, classified as either having developed diabetes (n = 1,878) or as being healthy (n = 3,080), as shown in Table 1. Approval for this study was provided by the Institutional Review Board of Chung Shan Medical University (IRB: CS1-23101).

### Variable definition

To ensure the integrity of our analysis, individuals diagnosed with T2D or any other form of diabetes at baseline were excluded from the study. Diabetes was defined according to established criteria: a fasting blood glucose level of ≥ 126 mg/dL, an HbA1c level of ≥ 6.5%, or self-reported physician-diagnosed diabetes based on participant questionnaires. Additionally, prediabetes was defined as a FBS level ranging from 100 to 125 mg/dL or an HbA1c level between 5.7% and 6.4%.

Lifestyle data, including exercise habits, were obtained through a comprehensive questionnaire administered by the TWB during the enrollment and follow-up stages. Individuals were classified as engaging in regular exercise if they exercised at least three times per week, with each session lasting 30 minutes or more, over the past three months. Exercise types considered in this analysis included a variety of physical activities, as defined by the TWB, encompassing aerobic exercises such as walking and jogging, anaerobic exercises like weight training and ball sports (including basketball and tennis), as well as traditional practices like Qigong.

The study participants were classified into four groups: (1) the “no exercise” group: included participants who had no excercise habit at both enrollment and follow-up; (2) the “no exercise to exercise” group: consisted of participants who did not have an exercise habit at enrollment but began exercising regularly during the follow-up period; (3) the “exercise to no exercise” group: comprised participants who had an exercise habit at enrollment but ceased exercising during the follow-up; and (4) the “exercise” group: included participants who maintained an exercise habit (i.e., exercising more than three times per week and at least 30 minutes each time) at both enrollment and follow-up. Additionally, the group labeled "no diabetes" referred to individuals who had prediabetes at enrollment but returned to a healthy state during follow-up. In contrast, the "diabetes" group included individuals who had prediabetes at enrollment and progressed to diabetes during follow-up. Our models included well-documented variables associated with diabetes, both inversely and positively, such as gender 19, age 20, smoking 21 alcohol consumption 22, waist-hip ratio/BMI 23, uric acid levels 24, coffee consumption 25, a vegetarian diet 26, follow-up duration, and hypertension and hyperlipidemia 27. Notably, the inclusion of coffee intake and a vegetarian diet as covariates reflects their significance in diabetes research. Specifically, studies have indicated that moderate coffee consumption may be linked to a reduced risk of T2D, potentially due to its antioxidant properties and effects on insulin sensitivity. Similarly, a vegetarian diet is associated with lower body weight and improved metabolic health, which are important factors in diabetes management.

### Statistical analysis

Statistical analysis was performed using the SAS 9.4 version (SAS Institute, Cary, NC, USA). Chi-square tests and ANOVA were used for demographic analysis to assess differences between categorical and continuous variables, respectively. Logistic regression models were used to examine the association between exercise status and diabetes in individuals with prediabetes. The significance level for the analysis was set at 0.05.

## Results

Table 1 presents the basic characteristics of the study population according to exercise status. There were 4,958 participants, with 2,891 women (58.31%) and 2,067 men (41.69%). Regarding the occurrence of diabetes, no statistically significant differences were observed among the exercise groups (P = 0.0701). In the "no exercise" group, there were 1,238 (61.59%) participants without diabetes and 772 (38.41%) participants with diabetes. Similarly, in the "no exercise to exercise" group, there were 425 (63.24%) participants without diabetes and 247 (36.76%) participants with diabetes. In the "exercise to no exercise" group, there were 310 (57.62%) participants without diabetes and 228 (42.38%) participants with diabetes. Lastly, in the "exercise" group, there were 1,107 (63.69%) participants without diabetes and 631 (36.31%) participants with diabetes. The mean age (± SE) for the study population was 51.980 years (± 0.224) in the "no exercise" group, 56.100 years (±0.390) in the "no exercise to exercise" group, and 56.831 years (± 0.437) in the "exercise" group. Other variables that differed among the various groups included BMI (P < 0.0001), uric acid (P = 0.0040), hypertension (P < 0.0001), hyperlipidemia (P = 0.0060), and follow-up duration (P < 0.0001).

Table 2 displays the logistic regression analysis examining the association between exercise status and diabetes in the prediabetes population. Among the various groups, only the "exercise" group demonstrated a significant association with a lower risk of diabetes (OR = 0.755; 95% CI = 0.640-0.892) compared to the "no exercise" group. However, the "no exercise to exercise" group (OR = 0.882; 95% CI = 0.716-1.087) and the "exercise to no exercise" group (OR = 0.994; 95% CI = 0.795-1.244) did not show a significant association with diabetes.

Table 3 presents the results of the association between exercise status and diabetes stratified by gender. Individuals who engaged in regular exercise were associated with a lower risk of diabetes, regardless of gender. The odds ratio (OR) for women was 0.752 (95% CI = 0.602-0.940), and for men, it was 0.762 (95% CI = 0.591-0.982). The "no exercise to exercise" group showed no significant association with diabetes in both women (OR = 1.010; 95% CI = 0.768-1.328) and men (OR = 0.722; 95% CI = 0.520-1.004). Furthermore, the "exercise to no exercise" group did not show a significant association with the progression to diabetes in either women (OR = 0.852; 95% CI = 0.629-1.153) or men (OR = 1.223; 95% CI = 0.869-1.723).

## Discussion

In the present study, the "exercise" group exhibited a significant association with a reduced risk of diabetes. Notably, transitioning from no exercise to exercise, as well as from exercise to no exercise, did not show significant associations with diabetes. Both women and men demonstrated a similar pattern regarding the prevalence of diabetes within the pre-diabetic population, indicating that regular exercise is linked to a decreased risk of diabetes, regardless of gender.

Previous research has highlighted the effectiveness of intensive lifestyle modifications, including dietary changes and regular exercise, in preventing the onset of diabetes in individuals with prediabetes 1. For instance, a study conducted in China collected data from 110,660 participants across 33 healthcare clinics between 1986 and 1992 9. Participants were randomly assigned to one of four groups: a control group or one of three active treatment groups—diet alone, exercise alone, or a combination of diet and exercise. Follow-up evaluations conducted at two-year intervals over six years revealed that the interventions led to reductions in diabetes risk by 31% (P < 0.03) for diet, 46% (P < 0.0005) for exercise, and 42% (P < 0.005) for the combined approach. Additionally, these interventions have also demonstrated favorable long-term results for cardiovascular and microvascular outcomes 28. However, most studies focused on the combination of diet and exercise.

The Diabetes Prevention Program (DPP) in the United States further reinforces our findings. This randomized clinical trial involved 27 centers and assessed whether lifestyle intervention or pharmacological therapy (metformin) could delay the onset of diabetes in individuals with impaired glucose tolerance (IGT) 29. In another study with nearly three years of follow-up, the diabetes incidence rate was 11 cases per 100 person-years in the control group, compared to 4.8 cases per 100 person-years in the lifestyle intervention group 10. Similarly, the Diabetes Prevention Study (DPS) in Finland, which involved 522 middle-aged subjects with impaired glucose tolerance, found a significant 58% reduction in diabetes risk for those in the exercise and diet intervention groups 11. Other findings from a health screening program in Japan with 458 male participants also demonstrated that intensive lifestyle interventions could significantly reduce the risk of developing type 2 diabetes 30. In India, a study involving 531 subjects found that lifestyle modifications (exercise and diet), metformin treatment, or a combination of both resulted in a relative risk reduction of 28.5%, 26.4%, and 28.2%, respectively, compared to the control group 31. Collectively, these studies consistently demonstrate that lifestyle interventions, particularly dietary modifications and increased physical activity, are associated with a reduced incidence of diabetes compared to placebo or usual care.

Prediabetes is often associated with several notable risk factors, such as excess weight or obesity, older age, sedentary lifestyle, unhealthy dietary patterns, and genetic predisposition 32. In our study, obesity and overweight (BMI ≥24) were linked to a higher risk of diabetes in the Taiwanese population. According to the 2005-2014 National Health and Nutrition Examination Survey (NHANES), over 80% of individuals diagnosed with prediabetes were either overweight or classified as obese (BMI ≥ 25) 33. While many randomized clinical trials have linked the effects of lifestyle modification primarily to weight loss 34, it is crucial to recognize that other factors may also play a significant role 9, 31.

Our findings revealed a similar trend in diabetes prevalence among the pre-diabetic population across both genders, indicating that regular exercise is crucial for reducing diabetes risk, regardless of gender. Research has identified gender disparities in the epidemiology of diabetes and obesity, likely stemming from a combination of cultural, biological, and lifestyle factors, as well as inequalities in treatment and prevention efforts 35, 36. Notably, men are diagnosed with diabetes at younger ages and BMI levels, while obesity—a significant risk factor—is generally more common in women 36. The biological variances may also include genetic predispositions that differ by gender 37. Furthermore, chronic inflammation, which is more pronounced in women, may interact with genetic predispositions and affect diabetes risk differently across genders.

Age was another significant risk factor for diabetes in our study. Prediabetes is common among older adults (over 60 years old); however, progression to diabetes in these individuals was found to be slower compared to those in middle age groups, based on a 6.5-year follow-up 38. As individuals age, various changes in body composition, insulin sensitivity, and glucose metabolism occur, which can elevate the risk of developing diabetes. Our study also demonstrated that subjects with hypertension and hyperlipidemia at enrollment exhibited a higher risk of developing diabetes. Previous data from the US NHANES (1988-2014) revealed a high prevalence of prediabetes among adults, with approximately half of these individuals having comorbidities such as hypertension or dyslipidemia, which significantly elevate the risk of cardiovascular and renal diseases 39.

It is important to note the limitations and strengths of our study. The TWB provides a large-scale, representative dataset covering over 95% of the Taiwanese population. However, the assessment of prediabetes was based solely on FBS and glycated hemoglobin (HbA1c) levels, without incorporating oral glucose tolerance tests that could provide a more comprehensive evaluation. Nevertheless, the definition of prediabetes used in this study aligns with recognized guidelines. Finally, physical activity or exercise data were only collected at baseline and the first follow-up, preventing the capture of fluctuations in participants' exercise habits throughout the study period. Consequently, participants with inconsistent exercise habits were classified based solely on their initial and final status without exclusion. This may affect the robustness of our findings, as participants with fluctuating exercise habits could have varying diabetes risk levels that are not fully captured in our analysis. Further research with more continuous monitoring of exercise habits could provide a clearer understanding of these dynamics.

## Conclusion

In summary, our study highlights that regular exercise is associated with a reduced risk of developing diabetes in individuals with prediabetes. These findings support the notion that lifestyle interventions, particularly exercise, are crucial in preventing the onset of diabetes. Gender and age did not significantly influence the association between exercise and diabetes risk in our study. However, further research is needed to understand the underlying mechanisms and develop targeted interventions for specific subgroups at high risk of diabetes. The promotion of lifestyle modifications, including exercise, should be integral to comprehensive diabetes prevention strategies.

## Figures and Tables

**Table 1:** Demographic characteristics of the study population based on exercise status

Variables	No exercise	No exercise to exercise	Exercise to no exercise	Exercise	P-value
	(n = 2010)	(n = 672)	(n = 538)	(n = 1738)	
Diabetes, n (%)					0.0701
No	1238 (61.59)	425 (63.24)	310 (57.62)	1107 (63.69)	
Yes	772 (38.41)	247 (36.76)	228 (42.38)	631 (36.31)	
Gender, n (%)					0.4505
Women	1177 (58.56)	408 (60.71)	313 (58.18)	993 (57.13)	
Men	833 (41.44)	264 (39.29)	225 (41.82)	745 (42.87)	
Age, years (mean ± SE)	51.980 ± 0.224	56.100 ± 0.390	56.831 ± 0.437	61.01 ± 0.195	<0.0001
Smoking, n (%)					0.3142
No	1556 (77.41)	516 (76.79)	417 (77.51)	1383 (79.57)	
Yes	454 (22.59)	156 (23.21)	121 (22.49)	355 (20.43)	
Alcohol consumption, n (%)					0.6200
No	1754 (87.26)	590 (87.80)	460 (85.50)	1505 (86.59)	
Yes	256 (12.74)	82 (12.20)	78 (14.50)	233 (13.41)	
Waist-hip ratio					0.5874
Normal (men ≤ 0.9; women ≤ 0.85)	789 (39.25)	280 (41.67)	221 (41.08)	713 (41.02)	
Abnormal (men >0.9; women >0.85)	1221 (60.75)	392 (58.33)	317 (58.92)	1025 (58.98)	
BMI categories, n (%)					<0.0001
Normal weight (18.5≤BMI<24 kg/m^2^)	763 (37.96)	299 (44.49)	199 (36.99)	855 (49.19)	
Underweight (BMI<18.5 kg/m^2^)	40 (1.99)	22 (3.27)	12 (2.23)	40 (2.30)	
Overweight (24≤BMI<27 kg/m^2^)	568 (28.26)	198 (29.46)	168 (31.23)	524 (30.15)	
Obesity (BMI≥27 kg/m^2^)	639 (31.79)	153 (22.77)	159 (29.55)	319 (18.35)	
Uric acid, n (%)					0.0040
Normal (women ≤ 6; men ≤ 7)	1541 (76.67)	536 (79.76)	413 (76.77)	1412 (81.24)	
Abnormal (women > 6; men > 7)	469 (23.33)	136 (20.24)	125 (23.23)	326 (18.76)	
Coffeeconsumption					0.7367
No	1127 (56.07)	374 (55.65)	291 (54.09)	987 (56.79)	
Yes	883 (43.93)	298 (44.35)	247 (45.91)	751 (43.21)	
Vegetarian diet					0.7552
No	1821 (90.60)	613 (91.22)	489 (90.89)	1592 (91.60)	
Yes	189 (9.40)	59 (8.78)	49 (9.11)	146 (8.40)	
Hypertension					<0.0001
No	1638 (81.49)	537 (79.91)	415 (77.14)	1301 (74.86)	
Yes	372 (18.51)	135 (20.09)	123 (22.86)	437 (25.14)	
Hyperlipidemia					0.0060
No	1761 (87.61)	590 (87.80)	452 (84.01)	1464 (84.23)	
Yes	249 (12.39)	82 (12.20)	86 (15.99)	274 (15.77)	
Follow-up duration	1632.380 ± 10.054	1656.110 ± 17.141	1651.340 ± 20.235	1536.030 ± 10.387	<0.0001

Abbreviations: n, sample size; %, percent; BMI, body mass index; kg, kilogram; m^2^, meter squared.

**Table 2:** The association between exercise status and diabetes risk in the pre-diabetes population

Variables	OR	95% CI	P-value
Exercise status (ref: no exercise)			
No exercise to exercise	0.882	0.716-1.087	0.2393
Exercise to no exercise	0.994	0.795-1.244	0.9584
Exercise	0.755	0.640-0.892	0.0010
Gender (ref: women)			
Men	0.944	0.803-1.110	0.4848
Age	1.046	1.038-1.054	<0.0001
Smoking (ref: No)			
Yes	1.245	1.028-1.508	0.0248
Alcohol consumption (ref: No)			
Yes	0.781	0.631-0.967	0.0233
Waist-hip ratio (ref: Normal)			
Abnormal	2.183	1.881-2.534	<0.0001
BMI categories (ref: Normal weight)			
Underweight	0.469	0.244-0.899	0.0227
Overweight	1.769	1.504-2.080	<0.0001
Obesity	3.945	3.295-4.724	<0.0001
Uric acid (ref: Normal)			
Abnormal	1.303	1.112-1.527	0.0011
Coffee consumption (ref: No)			
Yes	0.847	0.741-0.967	0.0142
Vegetarian diet (ref: No)			
Yes	1.003	0.796-1.263	0.9818
Hypertension (ref: No)			
Yes	1.640	1.391-1.933	<0.0001
Hyperlipidemia (ref: No)			
Yes	2.173	1.795-2.630	<0.0001
Follow-up duration	1.001	1.000-1.001	<0.0001

**Table 3:** The association between exercise status and progression of prediabetes to diabetes in men and women

Variables	Women			Men		
	OR	95% CI	P-value	OR	95% CI	P-value
Exercise status (ref: no exercise)						
No exercise to exercise	1.010	0.768-1.328	0.9439	0.722	0.520-1.004	0.0528
Exercise to no exercise	0.852	0.629-1.153	0.2987	1.223	0.869-1.723	0.2481
Exercise	0.752	0.602-0.940	0.0123	0.762	0.591-0.982	0.0359
Age	1.050	1.038-1.062	<0.0001	1.039	1.027-1.050	<0.0001
Smoking (ref: No)						
Yes	0.914	0.548-1.524	0.7293	1.329	1.078-1.640	0.0079
Alcohol consumption (ref: No)						
Yes	0.622	0.358-1.081	0.0921	0.827	0.653-1.046	0.1133
Waist-hip ratio (ref: Normal)						
Abnormal	1.984	1.632-2.412	<0.0001	2.523	1.992-3.194	<0.0001
BMI categories (ref: Normal weight)						
Underweight	0.471	0.228-0.970	0.0410	0.529	0.118-2.372	0.4052
Overweight	1.890	1.529-2.337	<0.0001	1.560	1.205-2.020	0.0007
Obesity	3.856	3.045-4.882	<0.0001	3.736	2.801-4.983	<0.0001
Uric acid (ref: Normal)						
Abnormal	1.994	1.584-2.510	<0.0001	0.857	0.685-1.073	0.1782
Coffee consumption (ref: No)						
Yes	0.926	0.774-1.107	0.3965	0.759	0.620-0.929	0.0076
Vegetarian diet (ref: No)						
Yes	1.109	0.832-1.477	0.4817	0.830	0.559-1.234	0.3581
Hypertension (ref: No)						
Yes	1.810	1.439-2.277	<0.0001	1.430	1.124-1.819	0.0036
Hyperlipidemia (ref: No)						
Yes	1.875	1.447-2.430	<0.0001	2.586	1.934-3.459	<0.0001
Follow-up duration	1.001	1.000-1.001	<0.0001	1.001	1.000-1.001	<0.0001
